# Duration of viral shedding and culture positivity with postvaccination SARS-CoV-2 delta variant infections

**DOI:** 10.1172/jci.insight.155483

**Published:** 2022-01-25

**Authors:** Mark J. Siedner, Julie Boucau, Rebecca F. Gilbert, Rockib Uddin, Jonathan Luu, Sebastien Haneuse, Tammy Vyas, Zahra Reynolds, Surabhi Iyer, Grace C. Chamberlin, Robert H. Goldstein, Crystal M. North, Chana A. Sacks, James Regan, James P. Flynn, Manish C. Choudhary, Jatin M. Vyas, Amy K. Barczak, Jacob E. Lemieux, Jonathan Z. Li

**Affiliations:** 1 Massachusetts General Hospital, Boston, Massachusetts, USA.; 2 Harvard Medical School, Boston, Massachusetts, USA.; 3 Ragon Institute of MGH, MIT and Harvard and; 4 Harvard T.H. Chan School of Public Health, Boston, Massachusetts, USA.; 5 Brigham and Women’s Hospital, Boston, Massachusetts, USA.; 6 Broad Institute, Cambridge, Massachusetts, USA

**Keywords:** COVID-19, Infectious disease, Epidemiology

## Abstract

Isolation guidelines for severe acute respiratory syndrome coronavirus 2 (SARS-CoV-2) are largely derived from data collected prior to the emergence of the delta variant. We followed a cohort of ambulatory patients with postvaccination breakthrough SARS-CoV-2 infections with longitudinal collection of nasal swabs for SARS-CoV-2 viral load quantification, whole-genome sequencing, and viral culture. All delta variant infections in our cohort were symptomatic, compared with 64% of non-delta variant infections. Symptomatic delta variant breakthrough infections were characterized by higher initial viral load, longer duration of virologic shedding by PCR, greater likelihood of replication-competent virus at early stages of infection, and longer duration of culturable virus compared with non-delta variants. The duration of time since vaccination was also correlated with both duration of PCR positivity and duration of detection of replication-competent virus. Nonetheless, no individuals with symptomatic delta variant infections had replication-competent virus by day 10 after symptom onset or 24 hours after resolution of symptoms. These data support US CDC isolation guidelines as of November 2021, which recommend isolation for 10 days or until symptom resolution and reinforce the importance of prompt testing and isolation among symptomatic individuals with delta breakthrough infections. Additional data are needed to evaluate these relationships among asymptomatic and more severe delta variant breakthrough infections.

## Graphical abstract

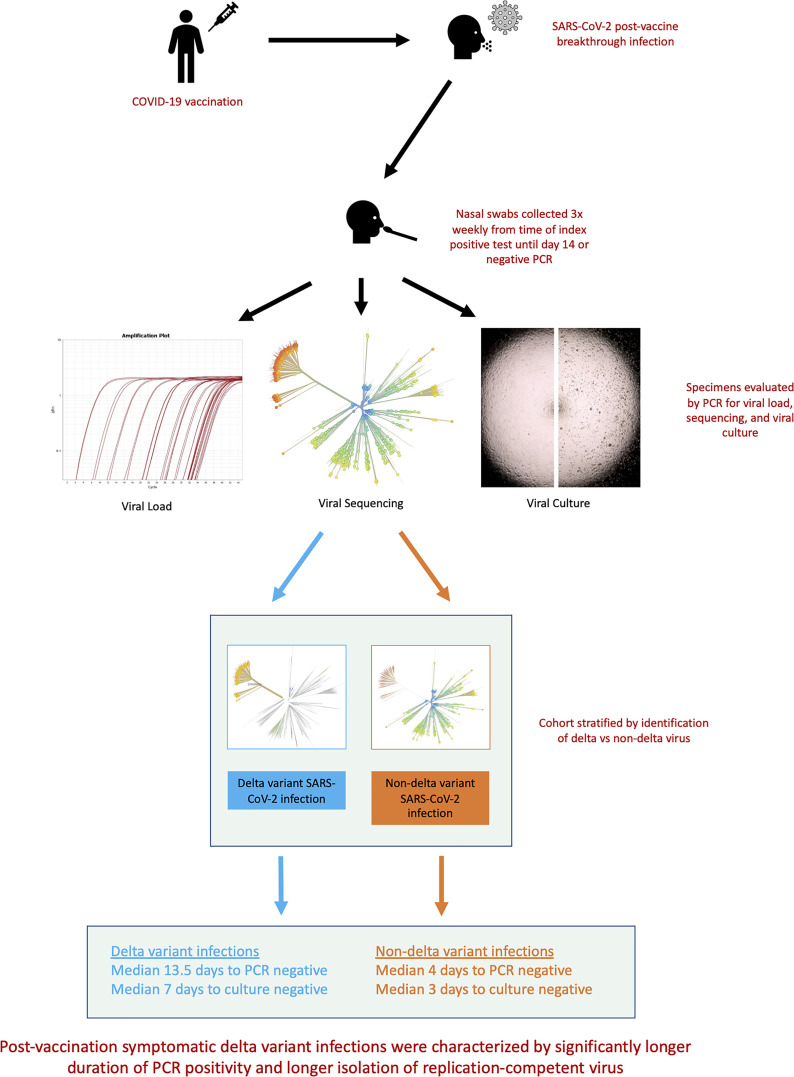

## Introduction

Isolation and distancing practices are fundamental elements of COVID-19 epidemic control. Guidelines authored by the US CDC as of November 2021 recommend that most individuals with severe acute respiratory syndrome coronavirus 2 (SARS-CoV-2) remain isolated for 10 days after a positive test (if asymptomatic) or 10 days from onset of symptoms and 1 day after resolution of symptoms (for symptomatic infections) (1). These guidelines were largely developed based on the low likelihood of recovering replication-competent virus after 10 days of symptoms for most patients (2–5), prior to the emergence of the delta variant as the dominant circulating strain globally (6). The delta variant has been associated with a higher basic reproductive number (7), higher viral loads at detection of infection (8), higher replication efficiency (9), and a shorter incubation period and generation time (10). However, there are few longitudinal data on delta variant infections that describe the duration of contagiousness or isolation of replication-competent virus, particularly with postvaccination breakthrough infections.

## Results

Twenty-four individuals with PCR-confirmed SARS-CoV-2 infection after vaccination were enrolled between January and August 2021 (Table 1). All 10 infections (42% of the total sample) after June 29, 2021, were confirmed by sequencing as delta variant infections. The 14 participants enrolled prior to that date had a diversity of variants, including alpha (*n* = 4), gamma (*n* = 1), and mu (*n* = 1). For 8 participants, the specimens contained insufficient virus for sequencing. All of these were collected when the delta variant constituted less than 10% of sequenced virus in Massachusetts and were presumed to be non-delta variant infections. Participants with delta and non-delta infections were similar in terms of age, sex, and COVID-19 vaccine manufacturer. Those with delta variant infections had a longer duration of time since completion of vaccination (median 160 vs. 29 days). Two individuals had an index positive PCR result between 3 and 4 weeks after their first vaccine dose but had not completed the second dose. Symptomatic infections were somewhat more likely with delta infections (100%) versus non-delta infections (64%, *P* = 0.053). Similarly, viral loads from the first study specimen were higher with delta infections (5.5 log_10_ copies/mL) compared with non-delta infections (2.0 log_10_ copies/mL, *P* = 0.005).

**Figure 1:**
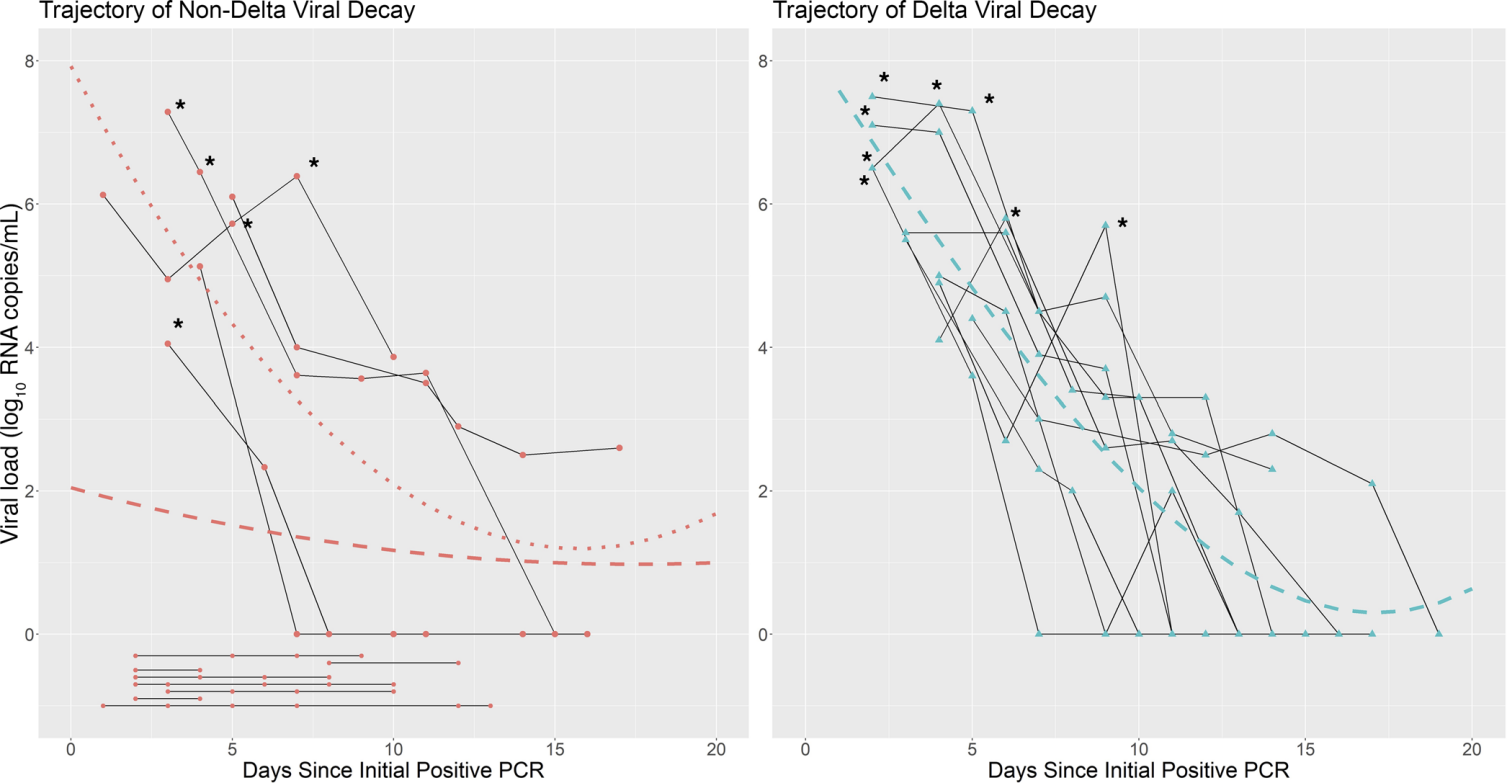
Viral load decay curve in individuals with postvaccination breakthrough SARS-CoV-2 non-delta and delta variant infections. Trajectory of viral load by PCR from time of index positive test for each study participant with non-delta variant (*n* = 14) (**A**) and delta variant (*n* = 8) (**B**) PCR-confirmed SARS-CoV-2 infection. Each connected solid line represents a participant. The dashed lines represent the total population mean line of fit derived from a regression equation, including quadratic and cubic terms. The dotted line represents a similar line of fit, but restricted to individuals who remained positive at day 3. Time points denoted with asterisks were viral culture positive.

**Figure 2:**
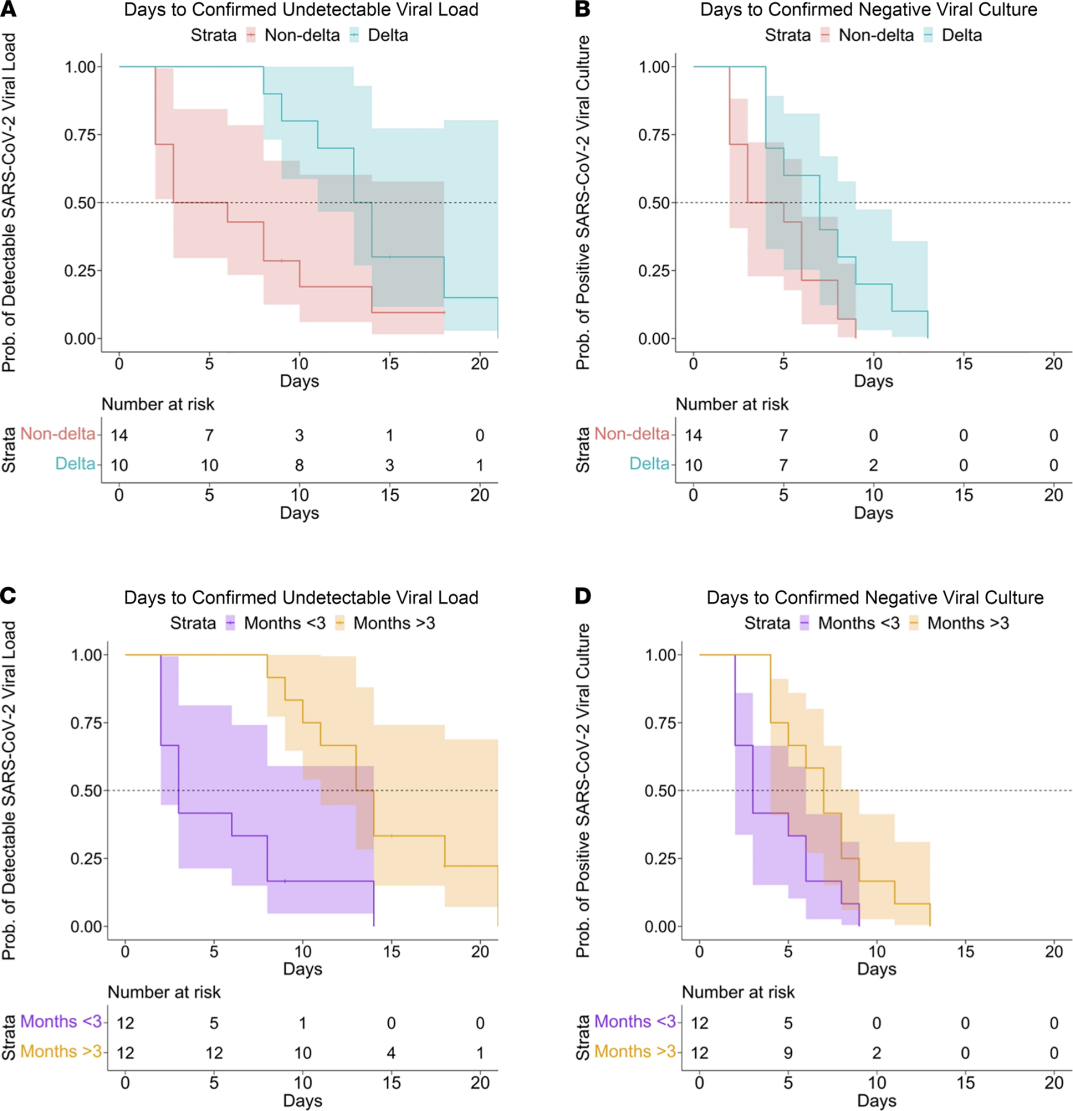
Kaplan-Meier survival curves demonstrating days from first positive PCR (or from symptom onset if earlier) to negative SARS-CoV-2 viral load and negative culture positivity among the entire sample. Survival curves for days to undetectable SARS-CoV-2 viral load (**A**) and viral culture (**B**) stratified by variant, and survival curves for days to undetectable SARS-CoV-2 viral load (**C**) and viral culture (**D**) stratified by duration since completion of COVID-19 vaccination. Observation time begins at the date of positive PCR for asymptomatic cases and date of symptom onset for symptomatic cases.

Delta variant postvaccine breakthrough infections were more likely to grow in culture than alternate variants infections (7 of 10 [70%] vs. 3 of 14 [23%] infections, *P* = 0.035). This pattern was consistent when restricted to symptomatic infections only (7 of 10 [70%] vs. 3 of 9 [33%] infections, *P* = 0.179). Individuals with delta variant infections had slower viral load decay, as assessed by PCR (median time, 13.5 vs. 4.5 days; HR, 0.38; 95% CI, 0.15, 0.95; Figure 1 and Figure 2A). Delta variant infection was also associated with lower hazard of conversion to negative viral culture (HR, 0.43; 95%, CI 0.18, 1.03), although the difference in median time to negative viral culture was less pronounced than it was for conversion to negative PCR (median time to negative viral load, 7 vs. 4 days; Figure 2B). Nine of 10 (90%) individuals with delta variant postvaccine breakthrough infections had a confirmed negative viral culture within 10 days of symptom onset. The remaining participant had culturable virus at day 11 but a negative culture at day 13. That participant remained symptomatic at day 11.

**Figure 3:**
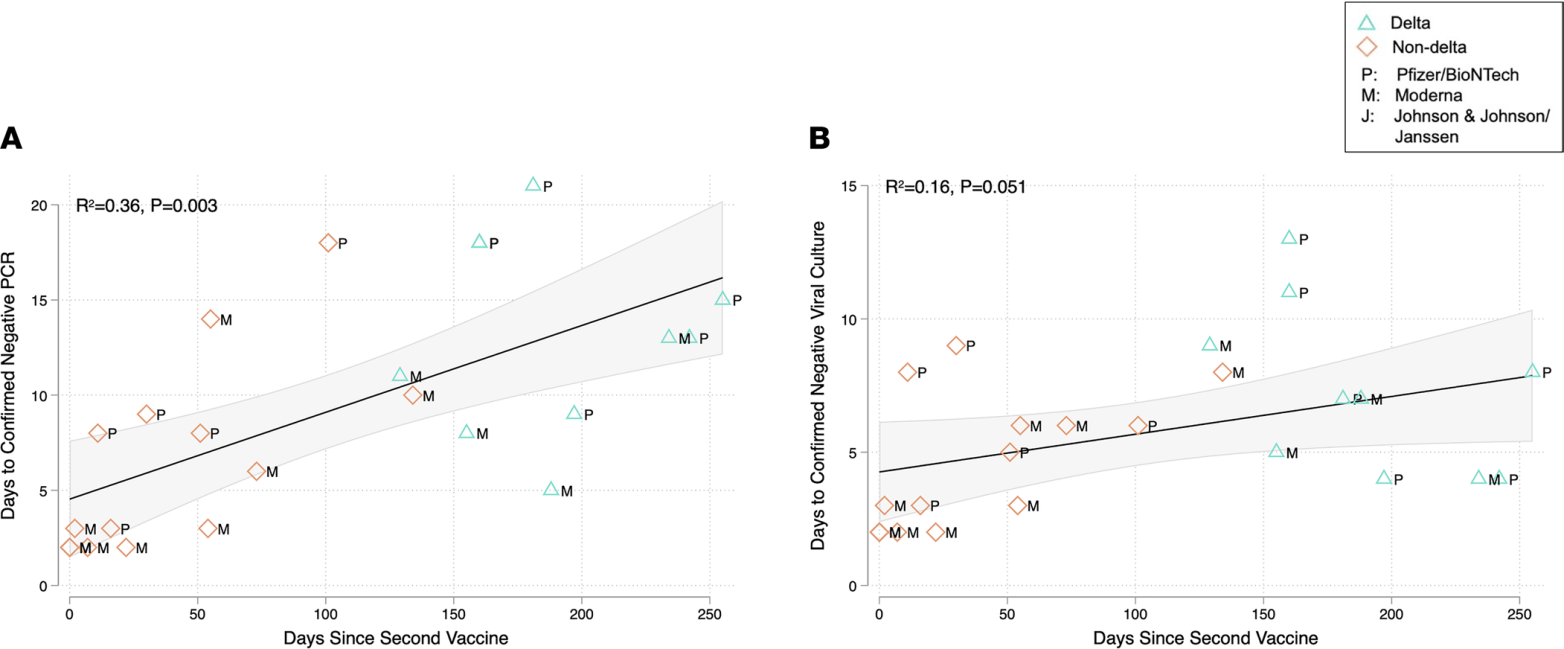
Scatter plots demonstrating the relationship between days since completion of COVID-19 vaccination and duration of SARS-CoV-2 PCR and culture positivity. (**A**) The relationship with duration of PCR positivity, and (**B**) the relationship for duration of culture positivity. Each point on the graph indicates an individual with postvaccination breakthrough COVID-19 infection (*n* = 24). Vaccines received are indicated by plot labels as Moderna (M), Pfizer/BioNTech (P), or Johnson & Johnson/Janssen (J). Black solid lines indicated the line of best fit from linear regression models, whereas the gray shaded areas indicate the 95% CI around this estimate. *R*^2^ and *P* values are estimates from these models. Green triangles indicate delta variant infections, whereas blue diamonds represent non-delta variant infections.

**Figure 4:**
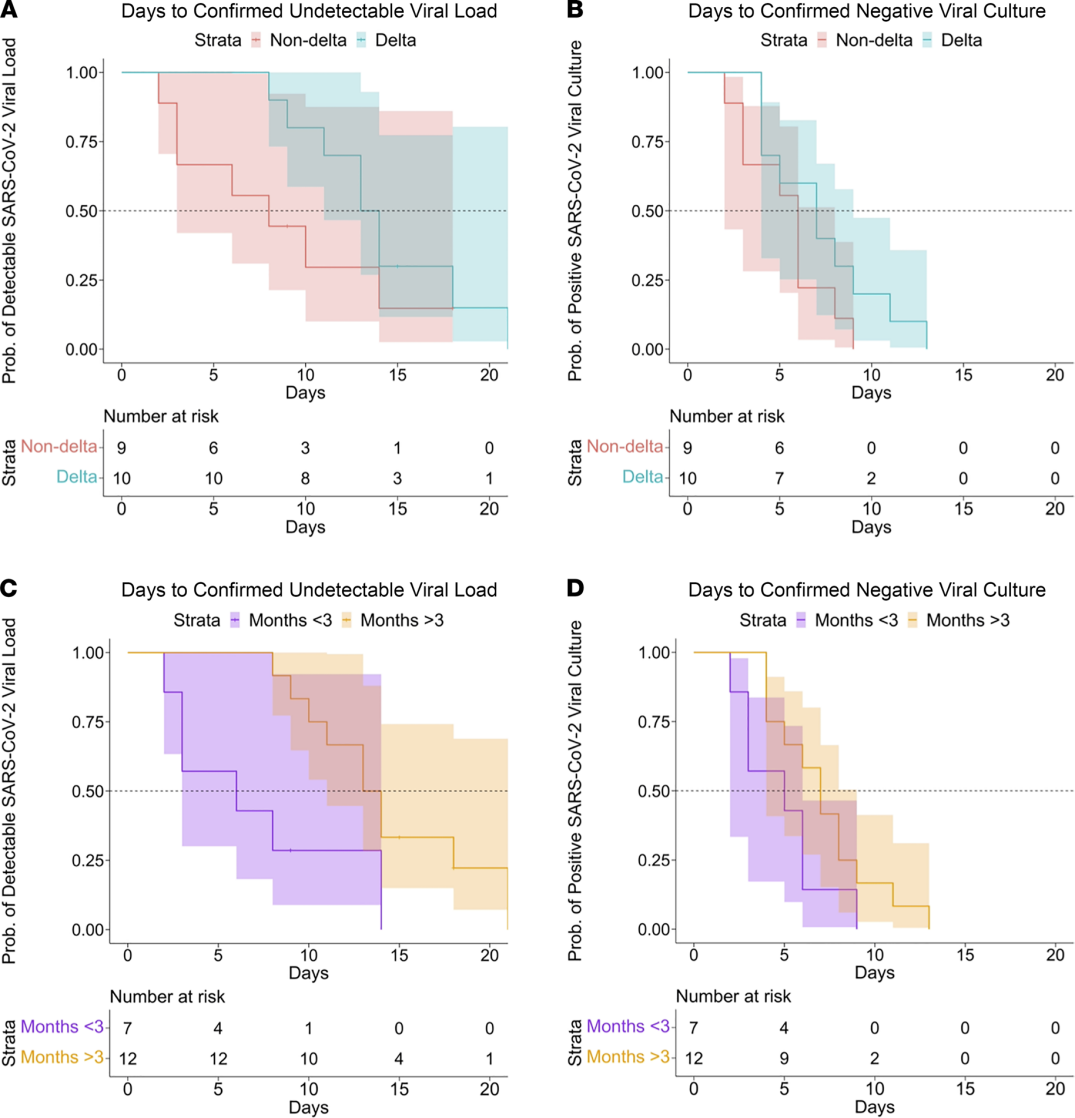
Kaplan-Meier survival curves demonstrating days from first symptom to negative SARS-CoV-2 viral load and negative culture positivity, restricted to the subsample with symptomatic infection. Survival curves for days to undetectable SARS-CoV-2 viral load (**A**) and viral culture (**B**) stratified by variant, and survival curves for days to undetectable SARS-CoV-2 viral load (**C**) and viral culture (**D**) stratified by duration since completion of COVID-19 vaccination.

Likewise, we found evidence that time to negative viral load by PCR was longer for individuals infected more than 3 months after completion of vaccination compared with those infected within 3 months of vaccination (median time, 13.5 vs. 3 days; HR, 0.20; 95% CI, 0.08, 0.53; Figure 2C), with a significant, albeit diminished, difference in time to negative viral culture (median time, 7 vs. 3 days; HR, 0.40; 95% CI, 0.17, 0.93; Figure 2D). When considering time from vaccination as a continuous measure, each additional 30 days since completion of vaccination was associated with an additional 1.3 days of PCR positivity (95% CI, 0.5, 2.0 days) and an additional 0.4 days of viral culture positivity (95% CI, –0.0, 0.9 days; Figure 3). Secondary analyses restricted to symptomatic individuals generally showed similar patterns with wider CIs due to the restricted sample size (Table 2 and Table 3 and Figure 4).

## Discussion

In this cohort of ambulatory individuals with postvaccination breakthrough infections, symptomatic delta variant SARS-CoV-2 infections were characterized by high initial viral load and a longer duration of virologic shedding, as detected by PCR (median, 13.5 days vs. 4.5 days). Moreover, identification of replication-competent virus by culture was more common with symptomatic delta variant infections (70%) than all non-delta infections (21%) and symptomatic non-delta infections (33%). Importantly, the duration of replication-competent virus was modestly prolonged among those with delta variant breakthrough infections (7 days vs. 4 days; HR, 0.43; 95% CI, 0.18, 1.03), a pattern which persisted when we restricted the analysis to symptomatic infections (7 days vs. 6 days; HR, 0.50; 95% CI 0.19, 1.32). Nonetheless, we detected only a single symptomatic delta breakthrough infection characterized by more than 10 days of replication-competent virus (11 days) in a participant who remained symptomatic on their final day of culture positivity (day 11).

In contrast to our findings, a prior study showed similar trajectories and duration of virologic shedding, as detected by PCR between delta and alpha viral variants, and shorter duration of shedding among delta variant infections (median, 6 vs. 13.5 days) (11). We suspect that this difference is explained by distinct features of our study populations. Whereas that former study included relatively young individuals being tested as part of their affiliation with a sports league, study participants with breakthrough delta infections in our study population were comparatively older adults, accessing SARS-CoV-2 testing through a healthcare system.

Our data are in keeping with CDC guidelines as of November 2021, which recommend isolation for 10 days or until symptom resolution for symptomatic postvaccination breakthrough infections. These results also reinforce that postvaccination breakthrough delta infections should be considered contagious and highlight the critical importance of prompt testing for symptomatic vaccinated individuals due to the high frequency of identification of replication-competent virus (5, 12).

Our study was limited to a small sample (*n* = 24) of vaccinated, ambulatory individuals with nonsevere infections. We were unable to sequence virus for 8 individuals in the study. However, all of these infections occurred before June 2021, when delta variant infections first constituted more than 10% of all infections in the region where the study occurred. We did not perform contact tracing in our study. Therefore, our conclusions about contagiousness are limited to inferences about the presence or absence of replication-competent virus but not confirmation of downstream infections from the cases detected in our study. All delta variant infections in our cohort were mild, but symptomatic, and thus our results should not be generalized to asymptomatic infections. Because of collinearity between delta variant infections and duration of time between vaccination and infection in our cohort, we cannot meaningfully distinguish the relative contributions of these two factors on transmission dynamics in breakthrough infections. Importantly, additional data are also needed to better elucidate the dynamics of delta variant infection in unvaccinated individuals. Work prior to the emergence of the delta variant suggests that the magnitude of viral load and duration of viral shedding are more prolonged among unvaccinated individuals (11, 13). That data, in combination with ours, which demonstrate longer shedding of nucleic acid and isolation of replication-competent virus in symptomatic delta variant-infected individuals, suggest that current isolation guidelines might not be adequate for all unvaccinated individuals with delta variant infections. Additional work is also needed to assess transmission dynamics in individuals with severe infection and after vaccine booster administration. Finally, we did not collect blood as part of this study, so we are unable to determine how host responses to vaccination contribute to virologic characteristics of breakthrough infections.

## Methods

### Study participants.

We enrolled nonhospitalized individuals with confirmed SARS-CoV-2 infection after vaccination. Participants were recruited after positive tests through one of two means. First, between January and March 2021, employees in the Mass General Brigham (Boston, Massachusetts, USA) Medical System were offered weekly COVID-19 testing irrespective of symptoms. Following the conclusion of that program, we began recruiting all individuals with positive SARS-CoV-2 PCR test results in the Mass General Brigham Medical System, which includes testing for symptomatic individuals as well as asymptomatic testing for contact tracing and screening procedures (e.g., preoperative clearance). All adults over 18 years of age who tested positive by either of these systems were eligible for inclusion in this study. For those who consented to participation, we conducted home visits 3 times weekly until negative PCR testing. At each visit, we obtained self-collected nasal swabs for SARS-CoV-2 PCR, culture, and whole-genome sequencing. Symptoms were assessed at each specimen collection and through medical chart review after study completion. Symptomatic infections were defined as those with COVID-19–related symptoms at any point during the observation period.

### Viral load quantification.

Viral load quantification and sequencing was conducted as previously reported (14). Briefly, we pelleted virions from nasal swab fluids after centrifugation at 21,000*g* for 2 hours at 4°C. We added TRIzol-LS Reagent (Thermo Fisher Scientific) to the pellets and incubated the pellets on ice after removing the supernatant. We then vortexed the pellets in 200 μL chloroform (MilliporeSigma), centrifuged the mixtures at 21,000*g* for 15 minutes at 4°C, removed the aqueous layer, and then treated the resulting solution with an equal volume of isopropanol (MilliporeSigma). We then added GlycoBlue Coprecipitant (Thermo Fisher Scientific) and 100 μL 3 M Sodium Acetate (Life Technologies) and incubated the mixtures in dry ice. We produced RNA pellets by centrifugation at 21,000*g* for 45 minutes at 4°C, discarded the supernatant, washed the RNA with cold 70% ethanol, and resuspended it in DEPC-treated water (Thermo Fisher Scientific). We quantified SARS-CoV-2 RNA virus using RT-qPCR with the US CDC 2019-nCoV_N1 primer and probe set (IDT) (15). Reactions included extracted RNA; 1X TaqPath 1-Step RT-qPCR Master Mix, CG (Thermo Fisher Scientific); forward and reverse primers; and the probe (15). We quantified viral copy numbers using N1 qPCR standards in 16-fold dilutions to generate standard curves. Each sample was run in triplicate with 2 nontemplate control wells that were included as negative controls. Additionally, we tested positive and negative controls alongside all samples. We assessed sample quality by quantifying the importin-8 (IPO8) housekeeping gene RNA level. Finally, to determine the efficiency of RNA extraction and qPCR amplification, we spiked (RCAS) (16) into each sample as an internal virion control.

### SARS-CoV-2 whole-genome sequencing.

We performed whole-genome sequencing using the Illumina COVIDSeq Test protocol. We constructed libraries using the Illumina Nextera XT Library Prep Kit and then pooled and quantified the libraries using a Qubit High Sensitivity dsDNA kit. Then, we performed genomic sequencing on an Illumina NextSeq 2000, Illumina NextSeq 550, or Illumina NovaSeq SP instrument. Sequences with an assembly length greater than 24000 base pairs were considered complete genomes, and we assigned those sequences a Pango lineage using the most up-to-date version of pangoLEARN assignment algorithm v2.4.2 (17).

All sequences were deposited in GenBank and GISAID. The samples were submitted to NCBI with Bioproject accession numbers PRNJA759255 and PRNJA622837.

### SARS-CoV-2 spike gene amplification.

We additionally performed spike gene amplification, as previously described (14), to determine variant types for specimens with low viral load to determine variants when whole-genome sequencing was unsuccessful. We used Superscript IV reverse transcriptase (Invitrogen) to conduct cDNA synthesis. To exclude PCR artifacts, we used two strategies to amplify the SARS-CoV-2 spike gene: (a) nested PCR amplification with in-house designed primer sets that targeted codon 1–814 of the spike gene and (b) the multiplexed primer pools designed with Primal Scheme generating 400 bp tiling amplicons based on the Arctic protocol (PCR round 1 primer sets, 21172-F, ACAGAACATTCTTGGAATGCT; 24021-R, TCTTCAATAAATGACCTCTTGC; PCR round 2 primer sets, 21361-F, AATCCAATTCAGTTGTCTTCC; 24002-R, TGCTTGGTTTTGATGGATCTG) (18). We separately pooled PCR products from both strategies and performed Illumina library construction using the Nextera XT Library Prep Kit (Illumina). We analyzed raw sequence data with PASeq v1.4 (https://www.paseq.org). We conducted data filtering with Trimmomatic (v0.30) (19), using a Q25/5 bp sliding window and a 70 bp minimum length. We filtered out nonviral contamination with BBsplit v35.76 (19). We then merged filtered reads using paired-end read merger v0.9.6 aligned to reference sequences with Bowtie2 v2.1.0) (20). Finally, amino acid variants were identified at the codon level with perl code and used to determine SARS-CoV-2 variant type.

### SARS-CoV-2 culture.

We performed viral culture as previously reported in the BSL3 laboratory of the Ragon Institute of MGH, MIT and Harvard (14, 21). Briefly, we detached Vero-E6 cells (ATCC) maintained in DMEM (Corning) supplemented with HEPES (Corning), 1X Penicillin (100 IU/mL)/Streptomycin (100 μg/mL) (Corning), 1X Glutamine (Glutamax, Thermo Fisher Scientific), and 10% FBS (MilliporeSigma) using Trypsin-EDTA (Thermo Fisher Scientific) and seeded the cells at 75,000 cells per well in 24-well plates or 20,000 cells per well in 96-well plates 16–20 hours before infection. We thawed specimens on ice, filtered the specimens through a Spin-X 0.45 μm filter (Corning) at 10,000*g* for 5 minutes, and diluted them 1:10 in DMEM supplemented with HEPES, 1X Penicillin/Streptomycin, and 1X Glutamine. We used 100 μL of the solution to inoculate triplicate wells in a 24-well plate. We then added 1 mL DMEM supplemented with HEPES, 1X Penicillin/Streptomycin, and 1X Glutamine and 2% FBS to each well after 1 hour of incubation and removed the viral inoculum. We added 25 μL of the undiluted filtrate to 4 wells of a 96-well plate and serial diluted (1:5) the filtrate in media containing 5 μg/mL polybrene (Santa Cruz Biotechnology). We centrifuged the 96-well plates for 1 hour at 2000*g* at 37°C. As a positive control, we used the SARS-CoV-2 isolate USA-WA1/2020 strain (BEI Resources). We observed viral culture plates at 3 and 7 days after infection with a light microscope and documented wells showing CPE. Finally, we harvested the supernatant of wells displaying CPE 10–14 days after infection and isolated RNA using a QIAamp Viral RNA Mini kit (QIAGEN) for confirmation of the viral sequence.

### Statistics.

We first evaluated patient-specific trajectories of quantitative viral load by PCR over time since index positive test by constructing spaghetti plots. To estimate grouped mean trajectories, stratified by variant, we fitted a linear regression model, including quadratic and cubic spline terms for time since the index positive PCR test (22). We then used the Kaplan-Meier estimator to estimate the survivor function for (a) time to negative viral by PCR testing and (b) time to negative viral culture. For both outcomes, we took the time of symptom onset (for symptomatic cases) or time of first positive PCR (for asymptomatic cases) as the origin of the timescale. We selected these definitions of left censoring to replicate US CDC isolation guidelines. For both outcomes, we estimated survivor functions stratified by delta versus non-delta variant infection and, separately, by duration of time from vaccination to infection, dichotomized as greater versus less than 90 days. We next fitted cox proportional hazards model with both outcomes and delta versus non-delta variant infection and, separately, time since completion of vaccination as predictors. In sensitivity analyses to assess for potential confounding by the presence of absence of symptoms, we reestimated Kaplan-Meier survivor functions after limiting the sample to individuals with symptomatic infection. Finally, we fit linear regression models and plotted scatter plots to assess relationships between duration of PCR and culture positivity and time since completion of vaccination as a continuous measure. *P* values of less than 0.05 were considered significant. Analyses were conducted with Stata (version 15.1) and R (version 4.0).

### Study approval.

Study procedures were reviewed and approved by the Human Subjects Institutional Review Board and the Institutional Biosafety Committee at Mass General Brigham. All participants gave verbal informed consent, as written consent was waived by the review committee based on the risk to benefit ratio of requiring in-person interactions for an observational study of COVID-19.

## Author contributions

MJS conceived the study design, participated in the data analysis, drafted the initial manuscript, and contributed editorial input. JB conducted experiments, participated in the data analysis, and contributed editorial input. RFG managed data collection, participated in data analysis, and contributed editorial input.RU participated in data collection and data analysis and contributed editorial input.JL, SH, TV, ZR, SI, GCC, RHG, CMN, and CAS participated in the data analysis and contributed editorial input.JR participated in the data collection, conducted experiments, and contributed editorial input.JPF conducted experiments and contributed editorial input.MCC conducted experiments, participated in the data analysis, and contributed editorial input. JMV conceived the study design, provided reagents, and contributed editorial input. AKB, JEL, and JZL conceived the study design, provided reagents, participated in the data analysis, and contributed editorial input. All authors approve of the final version of the manuscript.

## Figures and Tables

**Table 2:**
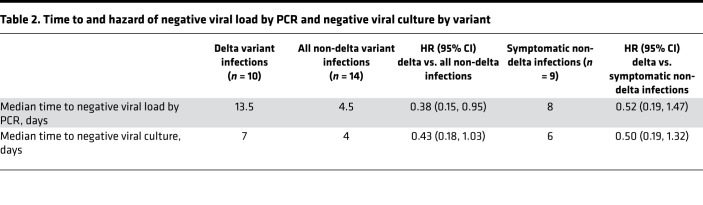
Time to and hazard of negative viral load by PCR and negative viral culture by variant

**Table 3:**
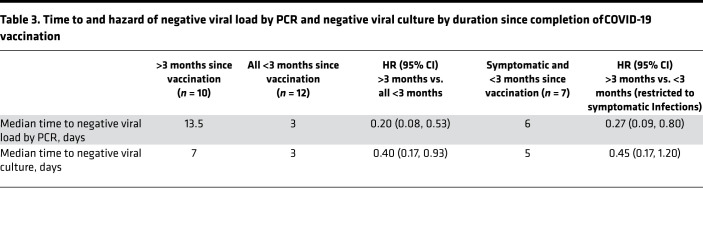
Time to and hazard of negative viral load by PCR and negative viral culture by duration since completion of COVID-19 vaccination

**Table 1:**
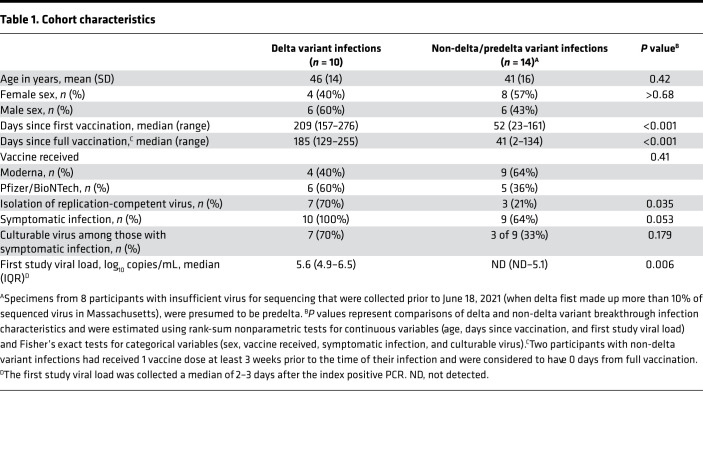
Cohort characteristics

## Version 1 12/06/2021

In-Press Preview

## Version 2 01/25/2022

Electronic publication
